# 2-Methyl-*N*-(4-methyl­benzo­yl)benzene­sulfonamide

**DOI:** 10.1107/S1600536810007440

**Published:** 2010-03-06

**Authors:** B. Thimme Gowda, Sabine Foro, P. A. Suchetan, Hartmut Fuess

**Affiliations:** a Department of Chemistry, Mangalore University, Mangalagangotri 574 199, Mangalore, India; b Institute of Materials Science, Darmstadt University of Technology, Petersenstrasse 23, D-64287 Darmstadt, Germany

## Abstract

The asymmetric unit of the title compound, C_15_H_15_NO_3_S, contains two independent mol­ecules. The conformations of the N—C bonds in the C—SO_2_—NH—C(O) segments have *gauche* torsions with respect to the SO bonds. Further, the mol­ecules are twisted at the *S* atoms with torsion angles of −53.1 (2) and 61.2 (2)° in the two mol­ecules. The dihedral angles between the sulfonyl benzene rings and the —SO_2_—NH—C—O segments are 86.0 (1) and 87.9 (1)°. Furthermore, the dihedral angles between the sulfonyl and the benzoyl benzene rings are 88.1 (1) and 83.5 (1)° in the two mol­ecules. In the crystal, mol­ecules are linked by N—H⋯O(S) hydrogen bonds.

## Related literature

For background to our study of the effect of ring and the side-chain substituents on the crystal structures of *N*-aromatic sulfonamides and for similar structures, see: Gowda *et al.* (2009 ▶; 2010 ▶); Suchetan *et al.* (2010 ▶).
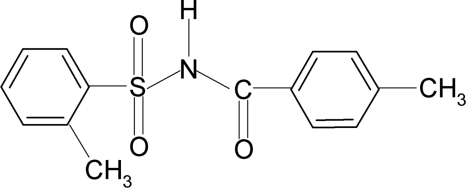


## Experimental

### 

#### Crystal data

C_15_H_15_NO_3_S
*M* *_r_* = 289.34
Triclinic, 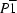

*a* = 10.9085 (8) Å
*b* = 12.1392 (9) Å
*c* = 12.3140 (9) Å
α = 118.846 (8)°
β = 95.965 (6)°
γ = 90.136 (6)°
*V* = 1417.98 (18) Å^3^
*Z* = 4
Mo *K*α radiation
μ = 0.23 mm^−1^
*T* = 299 K
0.48 × 0.44 × 0.12 mm

#### Data collection

Oxford Diffraction Xcalibur diffractometer with a Sapphire CCD Detector
Absorption correction: multi-scan (*CrysAlis RED*; Oxford Diffraction, 2009 ▶) *T*_min_ = 0.896, *T*_max_ = 0.972
9669 measured reflections
5139 independent reflections
4302 reflections with *I* > 2σ(*I*)
*R*_int_ = 0.013

#### Refinement

*R*[*F* ^2^ > 2σ(*F* ^2^)] = 0.037
*wR*(*F* ^2^) = 0.105
*S* = 1.05
5139 reflections
371 parameters
2 restraints
H atoms treated by a mixture of independent and constrained refinement
Δρ_max_ = 0.32 e Å^−3^
Δρ_min_ = −0.34 e Å^−3^

### 

Data collection: *CrysAlis CCD* (Oxford Diffraction, 2009 ▶); cell refinement: *CrysAlis RED* (Oxford Diffraction, 2009 ▶); data reduction: *CrysAlis RED*; program(s) used to solve structure: *SHELXS97* (Sheldrick, 2008 ▶); program(s) used to refine structure: *SHELXL97* (Sheldrick, 2008 ▶); molecular graphics: *PLATON* (Spek, 2009 ▶); software used to prepare material for publication: *SHELXL97*.

## Supplementary Material

Crystal structure: contains datablocks I, global. DOI: 10.1107/S1600536810007440/bq2199sup1.cif

Structure factors: contains datablocks I. DOI: 10.1107/S1600536810007440/bq2199Isup2.hkl

Additional supplementary materials:  crystallographic information; 3D view; checkCIF report

## Figures and Tables

**Table 1:** Hydrogen-bond geometry (Å, °)

*D*—H⋯*A*	*D*—H	H⋯*A*	*D*⋯*A*	*D*—H⋯*A*
N1—H1*N*⋯O4^i^	0.82 (2)	2.18 (2)	2.978 (2)	165 (2)
N2—H2*N*⋯O2^i^	0.83 (2)	2.20 (2)	3.022 (2)	171 (2)

Symmetry code: (i) 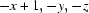.

**Fig. 1.**
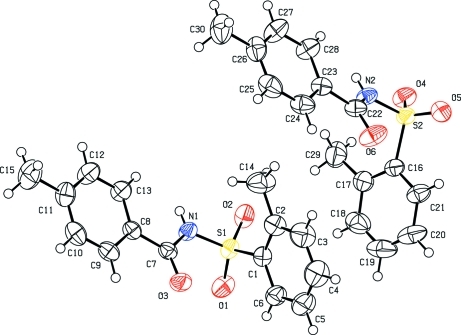
Molecular structure of the title compound, showing the atom-labelling scheme. Displacement ellipsoids are drawn at the 50% probability level.

**Fig. 2.**
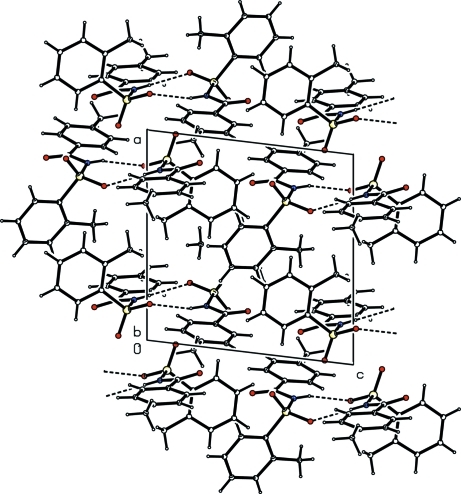
Molecular packing in the title compound. Hydrogen bonds are shown as dashed lines.

## supplementary crystallographic information

### Comment

Diaryl acylsulfonamides are known as potent antitumor agents against a broad spectrum of human tumor xenografts in nude mice. As a part of studying the effect of ring and the side chain substituents on the crystal structures of *N*-aromatic sulfonamides (Gowda *et al.*, 2009; 2010; Suchetan *et al.*, 2010), the structure of 2-methyl-*N*-(4-methylbenzoyl)benzenesulfonamide (I) has been determined. The asymmetric unit of the structure contains two independent molecules (Fig. 1). The conformations of the N—C bonds in the C—SO_2_—NH—C(O) segments have *gauche* torsions with respect to the SO bonds. Further, the conformations of the N—H bonds in the C—SO_2_—NH—C(O) segments are *anti* to the C=O bonds, similar to those observed in *N*-(benzoyl)benzenesulfonamide (II) (Gowda *et al.*, 2009), 2-methyl-*N*-(3-methylbenzoyl)benzenesulfonamide (III) (Gowda *et al.*, 2010) and *N*-(4-chlorobenzoyl)4-methyl- benzenesulfonamide (IV) (Suchetan *et al.*, 2010).

The molecules are twisted at the *S* atoms with the torsion angles of -53.1 (2)° and 61.2 (2)° in the two independent molecules. The dihedral angles between the sulfonyl benzene rings and the —SO_2_—NH—C—O segments are 86.0 (1)° (molecule 1) and 87.9 (1)° (molecule 2), compared to the values of 86.5 (1) in (II), 83.1 (1)° in (III), and 83.6 (1)° (molecule 1) and 81.0 (1)° (molecule 2) in (IV). Furthermore, the dihedral angles between the benzene rings are 88.1 (1)° (molecule 1) and 83.5 (1)° (molecule 2) in (I), compared to the values of 80.3 (1) in (II), 74.8 (1)° in (III), and 81.0 (1)° (molecule 1) and 76.3 (1)° (molecule 2) in (IV). The packing of molecules linked by of N—H···O(S) hydrogen bonds (Table 1) is shown in Fig. 2.

### Experimental

The title compound was prepared by refluxing a mixture of 4-methylbenzoic acid, 2-methylbenzenesulfonamide and phosphorous oxy chloride for 5 h on a water bath. The resultant mixture was cooled and poured into ice cold water. The solid, 2-methyl-*N*-(4-methylbenzoyl)benzenesulfonamide obtained was filtered, washed thoroughly with water and then dissolved in sodium bicarbonate solution. The compound was later reprecipitated by acidifying the filtered solution with dilute HCl. The filtered and dried compound was recrystallized to the constant melting point. Plate like colorless single crystals of the title compound used in X-ray diffraction studies were grown from a slow evaporation of its toluene solution at room temperature.

### Refinement

The H atoms of the NH groups were located in a difference map and later restrained to N—H = 0.86 (2) %A. The other H atoms were positioned with idealized geometry using a riding model with C—H = 0.93–0.96 Å All H atoms were refined with isotropic displacement parameters (set to 1.2 times of the *U*_eq_ of the parent atom).

### Crystal data

**Table:**

C_15_H_15_NO_3_S	*Z* = 4
*M**_r_* = 289.34	*F*(000) = 608
Triclinic, *P*1	*D*_x_ = 1.355 Mg m^−^^3^
Hall symbol: -P 1	Mo *K*α radiation, λ = 0.71073 Å
*a* = 10.9085 (8) Å	Cell parameters from 5092 reflections
*b* = 12.1392 (9) Å	θ = 2.5–27.9°
*c* = 12.3140 (9) Å	µ = 0.23 mm^−^^1^
α = 118.846 (8)°	*T* = 299 K
β = 95.965 (6)°	Plate, colourless
γ = 90.136 (6)°	0.48 × 0.44 × 0.12 mm
*V* = 1417.98 (18) Å^3^	

### Data collection

**Table:**

Oxford Diffraction Xcalibur diffractometer with a Sapphire CCD Detector	5139 independent reflections
Radiation source: fine-focus sealed tube	4302 reflections with *I* > 2σ(*I*)
graphite	*R*_int_ = 0.013
Rotation method data acquisition using ω and phi scans	θ_max_ = 25.4°, θ_min_ = 2.5°
Absorption correction: multi-scan (*CrysAlis RED*; Oxford Diffraction, 2009)	*h* = −10→13
*T*_min_ = 0.896, *T*_max_ = 0.972	*k* = −14→14
9669 measured reflections	*l* = −14→14

### Refinement

**Table:**

Refinement on *F*^2^	Primary atom site location: structure-invariant direct methods
Least-squares matrix: full	Secondary atom site location: difference Fourier map
*R*[*F*^2^ > 2σ(*F*^2^)] = 0.037	Hydrogen site location: inferred from neighbouring sites
*wR*(*F*^2^) = 0.105	H atoms treated by a mixture of independent and constrained refinement
*S* = 1.05	*w* = 1/[σ^2^(*F*_o_^2^) + (0.0505*P*)^2^ + 0.5865*P*] where *P* = (*F*_o_^2^ + 2*F*_c_^2^)/3
5139 reflections	(Δ/σ)_max_ = 0.020
371 parameters	Δρ_max_ = 0.32 e Å^−^^3^
2 restraints	Δρ_min_ = −0.34 e Å^−^^3^

### Special details

**Table:**

Geometry. All esds (except the esd in the dihedral angle between two l.s. planes) are estimated using the full covariance matrix. The cell esds are taken into account individually in the estimation of esds in distances, angles and torsion angles; correlations between esds in cell parameters are only used when they are defined by crystal symmetry. An approximate (isotropic) treatment of cell esds is used for estimating esds involving l.s. planes.
Refinement. Refinement of *F*^2^ against ALL reflections. The weighted *R*-factor wR and goodness of fit *S* are based on *F*^2^, conventional *R*-factors *R* are based on *F*, with *F* set to zero for negative *F*^2^. The threshold expression of *F*^2^ > σ(*F*^2^) is used only for calculating *R*-factors(gt) etc. and is not relevant to the choice of reflections for refinement. *R*-factors based on *F*^2^ are statistically about twice as large as those based on *F*, and *R*- factors based on ALL data will be even larger.

### Fractional atomic coordinates and isotropic or equivalent isotropic displacement parameters (Å^2^)

**Table:**

	*x*	*y*	*z*	*U*_iso_*/*U*_eq_	
S1	0.26147 (4)	0.15226 (4)	0.32500 (4)	0.03838 (13)	
O1	0.18593 (13)	0.11786 (14)	0.39241 (14)	0.0545 (4)	
O2	0.29413 (13)	0.05502 (12)	0.20811 (12)	0.0494 (4)	
O3	0.19313 (14)	0.40481 (14)	0.48858 (12)	0.0534 (4)	
N1	0.19457 (15)	0.25319 (14)	0.28899 (14)	0.0391 (4)	
H1N	0.1743 (19)	0.2237 (19)	0.2135 (15)	0.047*	
C1	0.39707 (17)	0.23373 (17)	0.42492 (17)	0.0390 (4)	
C2	0.49086 (19)	0.2806 (2)	0.38683 (19)	0.0498 (5)	
C3	0.5950 (2)	0.3360 (2)	0.4727 (2)	0.0669 (7)	
H3	0.6600	0.3679	0.4507	0.080*	
C4	0.6055 (2)	0.3455 (3)	0.5890 (2)	0.0717 (7)	
H4	0.6770	0.3829	0.6439	0.086*	
C5	0.5115 (2)	0.3006 (2)	0.6245 (2)	0.0653 (6)	
H5	0.5187	0.3074	0.7035	0.078*	
C6	0.4062 (2)	0.2453 (2)	0.54305 (18)	0.0487 (5)	
H6	0.3412	0.2157	0.5671	0.058*	
C7	0.16838 (17)	0.37305 (17)	0.37906 (16)	0.0381 (4)	
C8	0.11559 (17)	0.45739 (16)	0.33317 (16)	0.0374 (4)	
C9	0.07548 (18)	0.57174 (18)	0.42108 (19)	0.0453 (5)	
H9	0.0799	0.5910	0.5043	0.054*	
C10	0.0294 (2)	0.65630 (18)	0.3854 (2)	0.0524 (5)	
H10	0.0013	0.7314	0.4448	0.063*	
C11	0.0240 (2)	0.63239 (19)	0.2635 (2)	0.0528 (5)	
C12	0.0645 (2)	0.5187 (2)	0.1765 (2)	0.0560 (6)	
H12	0.0623	0.5010	0.0939	0.067*	
C13	0.1078 (2)	0.43154 (18)	0.20990 (18)	0.0468 (5)	
H13	0.1321	0.3548	0.1494	0.056*	
C14	0.4853 (3)	0.2737 (3)	0.2615 (2)	0.0750 (8)	
H14A	0.4184	0.3208	0.2521	0.090*	
H14B	0.4724	0.1874	0.1971	0.090*	
H14C	0.5616	0.3086	0.2552	0.090*	
C15	−0.0234 (3)	0.7268 (2)	0.2253 (3)	0.0799 (8)	
H15A	0.0410	0.7900	0.2455	0.096*	
H15B	−0.0925	0.7657	0.2690	0.096*	
H15C	−0.0489	0.6846	0.1369	0.096*	
S2	0.86754 (5)	−0.02893 (4)	0.09718 (4)	0.03994 (14)	
O4	0.82660 (15)	−0.14503 (12)	−0.01285 (12)	0.0534 (4)	
O5	0.99533 (14)	−0.00545 (16)	0.13898 (14)	0.0603 (4)	
O6	0.87360 (17)	0.24170 (13)	0.25506 (13)	0.0618 (4)	
N2	0.81576 (16)	0.07925 (14)	0.06276 (14)	0.0394 (4)	
H2N	0.7862 (19)	0.0503 (19)	−0.0110 (15)	0.047*	
C16	0.78770 (19)	−0.01250 (16)	0.22121 (16)	0.0394 (4)	
C17	0.6610 (2)	−0.04204 (19)	0.2040 (2)	0.0492 (5)	
C18	0.6095 (3)	−0.0268 (2)	0.3090 (3)	0.0672 (7)	
H18	0.5252	−0.0448	0.3021	0.081*	
C19	0.6791 (3)	0.0136 (2)	0.4219 (2)	0.0740 (8)	
H19	0.6416	0.0225	0.4899	0.089*	
C20	0.8024 (3)	0.0410 (2)	0.4359 (2)	0.0670 (7)	
H20	0.8491	0.0672	0.5128	0.080*	
C21	0.8582 (2)	0.02962 (18)	0.33558 (18)	0.0504 (5)	
H21	0.9422	0.0501	0.3448	0.060*	
C22	0.82979 (18)	0.20707 (17)	0.14844 (17)	0.0410 (4)	
C23	0.78477 (18)	0.29425 (17)	0.10290 (17)	0.0398 (4)	
C24	0.7648 (2)	0.41595 (19)	0.1922 (2)	0.0531 (5)	
H24	0.7803	0.4398	0.2766	0.064*	
C25	0.7219 (2)	0.5015 (2)	0.1563 (2)	0.0610 (6)	
H25	0.7076	0.5823	0.2173	0.073*	
C26	0.6995 (2)	0.4704 (2)	0.0320 (2)	0.0567 (6)	
C27	0.7227 (2)	0.3500 (2)	−0.0563 (2)	0.0609 (6)	
H27	0.7106	0.3276	−0.1404	0.073*	
C28	0.7637 (2)	0.26236 (19)	−0.02221 (19)	0.0513 (5)	
H28	0.7772	0.1814	−0.0834	0.062*	
C29	0.5796 (2)	−0.0874 (3)	0.0826 (2)	0.0685 (7)	
H29A	0.5912	−0.0308	0.0502	0.082*	
H29B	0.4949	−0.0901	0.0962	0.082*	
H29C	0.6008	−0.1702	0.0238	0.082*	
C30	0.6541 (3)	0.5661 (3)	−0.0056 (3)	0.0815 (8)	
H30A	0.5804	0.5998	0.0317	0.098*	
H30B	0.6365	0.5262	−0.0949	0.098*	
H30C	0.7166	0.6331	0.0225	0.098*	

### Atomic displacement parameters (Å^2^)

**Table:**

	*U*^11^	*U*^22^	*U*^33^	*U*^12^	*U*^13^	*U*^23^
S1	0.0424 (3)	0.0321 (2)	0.0363 (2)	−0.00049 (18)	−0.00347 (19)	0.01476 (19)
O1	0.0526 (9)	0.0604 (9)	0.0576 (9)	−0.0133 (7)	−0.0069 (7)	0.0368 (8)
O2	0.0572 (9)	0.0328 (7)	0.0423 (7)	0.0068 (6)	−0.0058 (6)	0.0078 (6)
O3	0.0694 (10)	0.0543 (9)	0.0313 (7)	0.0173 (7)	0.0115 (6)	0.0156 (6)
N1	0.0493 (9)	0.0338 (8)	0.0276 (8)	0.0066 (7)	−0.0003 (7)	0.0106 (7)
C1	0.0397 (10)	0.0329 (9)	0.0374 (10)	0.0011 (7)	−0.0001 (8)	0.0123 (8)
C2	0.0490 (12)	0.0469 (11)	0.0472 (11)	−0.0026 (9)	0.0048 (9)	0.0182 (9)
C3	0.0514 (13)	0.0710 (16)	0.0677 (16)	−0.0165 (12)	−0.0002 (11)	0.0269 (13)
C4	0.0541 (14)	0.0795 (17)	0.0605 (15)	−0.0170 (12)	−0.0168 (12)	0.0221 (13)
C5	0.0617 (15)	0.0782 (17)	0.0444 (12)	−0.0048 (12)	−0.0100 (11)	0.0238 (12)
C6	0.0490 (12)	0.0526 (12)	0.0400 (11)	−0.0006 (9)	−0.0003 (9)	0.0201 (9)
C7	0.0394 (10)	0.0374 (10)	0.0324 (10)	0.0041 (8)	0.0083 (8)	0.0121 (8)
C8	0.0383 (10)	0.0315 (9)	0.0358 (9)	0.0022 (7)	0.0060 (7)	0.0108 (8)
C9	0.0480 (11)	0.0370 (10)	0.0410 (10)	0.0031 (8)	0.0119 (9)	0.0099 (8)
C10	0.0547 (12)	0.0299 (10)	0.0612 (14)	0.0085 (9)	0.0158 (10)	0.0113 (9)
C11	0.0576 (13)	0.0351 (10)	0.0616 (14)	0.0066 (9)	0.0018 (10)	0.0213 (10)
C12	0.0790 (16)	0.0432 (11)	0.0425 (11)	0.0102 (11)	0.0011 (11)	0.0192 (9)
C13	0.0642 (13)	0.0321 (10)	0.0360 (10)	0.0107 (9)	0.0056 (9)	0.0100 (8)
C14	0.0736 (17)	0.093 (2)	0.0635 (16)	−0.0173 (15)	0.0087 (13)	0.0424 (15)
C15	0.099 (2)	0.0528 (14)	0.089 (2)	0.0193 (14)	0.0012 (16)	0.0377 (14)
S2	0.0519 (3)	0.0363 (2)	0.0295 (2)	0.0112 (2)	0.00847 (19)	0.01357 (19)
O4	0.0870 (11)	0.0333 (7)	0.0325 (7)	0.0155 (7)	0.0102 (7)	0.0095 (6)
O5	0.0500 (9)	0.0811 (11)	0.0515 (9)	0.0161 (8)	0.0107 (7)	0.0325 (8)
O6	0.1017 (13)	0.0407 (8)	0.0329 (8)	−0.0081 (8)	−0.0062 (8)	0.0127 (6)
N2	0.0574 (10)	0.0313 (8)	0.0256 (7)	0.0011 (7)	0.0023 (7)	0.0112 (6)
C16	0.0574 (12)	0.0287 (9)	0.0347 (9)	0.0100 (8)	0.0114 (8)	0.0164 (8)
C17	0.0594 (13)	0.0407 (11)	0.0552 (12)	0.0117 (9)	0.0148 (10)	0.0277 (10)
C18	0.0759 (17)	0.0620 (15)	0.0802 (18)	0.0143 (12)	0.0339 (14)	0.0427 (14)
C19	0.117 (2)	0.0593 (15)	0.0607 (16)	0.0138 (15)	0.0431 (16)	0.0346 (13)
C20	0.113 (2)	0.0522 (13)	0.0367 (12)	0.0010 (14)	0.0115 (13)	0.0217 (10)
C21	0.0743 (15)	0.0393 (10)	0.0363 (10)	0.0023 (10)	0.0055 (10)	0.0176 (9)
C22	0.0527 (11)	0.0329 (9)	0.0318 (10)	−0.0041 (8)	0.0071 (8)	0.0110 (8)
C23	0.0464 (11)	0.0326 (9)	0.0383 (10)	−0.0030 (8)	0.0080 (8)	0.0151 (8)
C24	0.0708 (14)	0.0391 (11)	0.0442 (11)	0.0037 (10)	0.0172 (10)	0.0141 (9)
C25	0.0727 (16)	0.0378 (11)	0.0713 (16)	0.0128 (10)	0.0285 (13)	0.0216 (11)
C26	0.0493 (12)	0.0495 (12)	0.0794 (16)	0.0028 (10)	0.0068 (11)	0.0377 (12)
C27	0.0815 (17)	0.0501 (13)	0.0525 (13)	−0.0046 (11)	−0.0090 (12)	0.0293 (11)
C28	0.0741 (15)	0.0347 (10)	0.0398 (11)	−0.0019 (10)	−0.0001 (10)	0.0153 (9)
C29	0.0557 (14)	0.0772 (17)	0.0816 (18)	0.0049 (12)	0.0001 (12)	0.0473 (15)
C30	0.0750 (18)	0.0709 (17)	0.119 (2)	0.0144 (14)	0.0097 (16)	0.0620 (18)

### Geometric parameters (Å, °)

**Table:**

S1—O1	1.4204 (15)	S2—O5	1.4131 (16)
S1—O2	1.4357 (14)	S2—O4	1.4318 (14)
S1—N1	1.6407 (16)	S2—N2	1.6474 (16)
S1—C1	1.7665 (18)	S2—C16	1.7648 (18)
O3—C7	1.209 (2)	O6—C22	1.210 (2)
N1—C7	1.393 (2)	N2—C22	1.389 (2)
N1—H1N	0.823 (15)	N2—H2N	0.826 (15)
C1—C6	1.385 (3)	C16—C21	1.384 (3)
C1—C2	1.394 (3)	C16—C17	1.395 (3)
C2—C3	1.386 (3)	C17—C18	1.395 (3)
C2—C14	1.500 (3)	C17—C29	1.501 (3)
C3—C4	1.372 (4)	C18—C19	1.368 (4)
C3—H3	0.9300	C18—H18	0.9300
C4—C5	1.364 (4)	C19—C20	1.358 (4)
C4—H4	0.9300	C19—H19	0.9300
C5—C6	1.372 (3)	C20—C21	1.381 (3)
C5—H5	0.9300	C20—H20	0.9300
C6—H6	0.9300	C21—H21	0.9300
C7—C8	1.481 (3)	C22—C23	1.481 (3)
C8—C13	1.387 (3)	C23—C24	1.387 (3)
C8—C9	1.393 (2)	C23—C28	1.388 (3)
C9—C10	1.375 (3)	C24—C25	1.376 (3)
C9—H9	0.9300	C24—H24	0.9300
C10—C11	1.379 (3)	C25—C26	1.382 (3)
C10—H10	0.9300	C25—H25	0.9300
C11—C12	1.386 (3)	C26—C27	1.381 (3)
C11—C15	1.508 (3)	C26—C30	1.511 (3)
C12—C13	1.376 (3)	C27—C28	1.379 (3)
C12—H12	0.9300	C27—H27	0.9300
C13—H13	0.9300	C28—H28	0.9300
C14—H14A	0.9600	C29—H29A	0.9600
C14—H14B	0.9600	C29—H29B	0.9600
C14—H14C	0.9600	C29—H29C	0.9600
C15—H15A	0.9600	C30—H30A	0.9600
C15—H15B	0.9600	C30—H30B	0.9600
C15—H15C	0.9600	C30—H30C	0.9600
			
O1—S1—O2	118.37 (9)	O5—S2—O4	118.33 (9)
O1—S1—N1	110.91 (9)	O5—S2—N2	110.40 (9)
O2—S1—N1	103.89 (8)	O4—S2—N2	103.58 (8)
O1—S1—C1	108.05 (9)	O5—S2—C16	108.84 (9)
O2—S1—C1	109.46 (9)	O4—S2—C16	109.58 (9)
N1—S1—C1	105.41 (8)	N2—S2—C16	105.28 (8)
C7—N1—S1	122.55 (13)	C22—N2—S2	122.31 (13)
C7—N1—H1N	124.3 (15)	C22—N2—H2N	124.1 (15)
S1—N1—H1N	113.0 (15)	S2—N2—H2N	113.5 (15)
C6—C1—C2	121.80 (18)	C21—C16—C17	122.16 (19)
C6—C1—S1	115.72 (15)	C21—C16—S2	116.18 (16)
C2—C1—S1	122.45 (15)	C17—C16—S2	121.65 (15)
C3—C2—C1	116.1 (2)	C18—C17—C16	116.0 (2)
C3—C2—C14	119.5 (2)	C18—C17—C29	119.4 (2)
C1—C2—C14	124.38 (19)	C16—C17—C29	124.64 (19)
C4—C3—C2	122.3 (2)	C19—C18—C17	122.0 (3)
C4—C3—H3	118.9	C19—C18—H18	119.0
C2—C3—H3	118.9	C17—C18—H18	119.0
C5—C4—C3	120.4 (2)	C20—C19—C18	120.8 (2)
C5—C4—H4	119.8	C20—C19—H19	119.6
C3—C4—H4	119.8	C18—C19—H19	119.6
C4—C5—C6	119.6 (2)	C19—C20—C21	119.8 (2)
C4—C5—H5	120.2	C19—C20—H20	120.1
C6—C5—H5	120.2	C21—C20—H20	120.1
C5—C6—C1	119.8 (2)	C20—C21—C16	119.3 (2)
C5—C6—H6	120.1	C20—C21—H21	120.4
C1—C6—H6	120.1	C16—C21—H21	120.4
O3—C7—N1	119.72 (17)	O6—C22—N2	119.92 (17)
O3—C7—C8	123.46 (16)	O6—C22—C23	123.46 (17)
N1—C7—C8	116.76 (15)	N2—C22—C23	116.58 (16)
C13—C8—C9	118.46 (18)	C24—C23—C28	118.56 (19)
C13—C8—C7	124.17 (16)	C24—C23—C22	117.17 (17)
C9—C8—C7	117.33 (17)	C28—C23—C22	124.26 (17)
C10—C9—C8	120.27 (19)	C25—C24—C23	120.2 (2)
C10—C9—H9	119.9	C25—C24—H24	119.9
C8—C9—H9	119.9	C23—C24—H24	119.9
C9—C10—C11	121.55 (18)	C24—C25—C26	121.7 (2)
C9—C10—H10	119.2	C24—C25—H25	119.1
C11—C10—H10	119.2	C26—C25—H25	119.1
C10—C11—C12	117.92 (19)	C27—C26—C25	117.7 (2)
C10—C11—C15	121.4 (2)	C27—C26—C30	121.3 (2)
C12—C11—C15	120.6 (2)	C25—C26—C30	121.0 (2)
C13—C12—C11	121.3 (2)	C28—C27—C26	121.4 (2)
C13—C12—H12	119.3	C28—C27—H27	119.3
C11—C12—H12	119.3	C26—C27—H27	119.3
C12—C13—C8	120.41 (18)	C27—C28—C23	120.39 (19)
C12—C13—H13	119.8	C27—C28—H28	119.8
C8—C13—H13	119.8	C23—C28—H28	119.8
C2—C14—H14A	109.5	C17—C29—H29A	109.5
C2—C14—H14B	109.5	C17—C29—H29B	109.5
H14A—C14—H14B	109.5	H29A—C29—H29B	109.5
C2—C14—H14C	109.5	C17—C29—H29C	109.5
H14A—C14—H14C	109.5	H29A—C29—H29C	109.5
H14B—C14—H14C	109.5	H29B—C29—H29C	109.5
C11—C15—H15A	109.5	C26—C30—H30A	109.5
C11—C15—H15B	109.5	C26—C30—H30B	109.5
H15A—C15—H15B	109.5	H30A—C30—H30B	109.5
C11—C15—H15C	109.5	C26—C30—H30C	109.5
H15A—C15—H15C	109.5	H30A—C30—H30C	109.5
H15B—C15—H15C	109.5	H30B—C30—H30C	109.5
			
O1—S1—N1—C7	63.65 (17)	O5—S2—N2—C22	−56.08 (17)
O2—S1—N1—C7	−168.15 (15)	O4—S2—N2—C22	176.27 (15)
C1—S1—N1—C7	−53.06 (17)	C16—S2—N2—C22	61.22 (17)
O1—S1—C1—C6	1.77 (18)	O5—S2—C16—C21	5.46 (17)
O2—S1—C1—C6	−128.41 (15)	O4—S2—C16—C21	136.28 (15)
N1—S1—C1—C6	120.41 (15)	N2—S2—C16—C21	−112.89 (15)
O1—S1—C1—C2	−179.98 (16)	O5—S2—C16—C17	−173.72 (15)
O2—S1—C1—C2	49.84 (19)	O4—S2—C16—C17	−42.90 (17)
N1—S1—C1—C2	−61.34 (18)	N2—S2—C16—C17	67.92 (17)
C6—C1—C2—C3	1.8 (3)	C21—C16—C17—C18	−0.1 (3)
S1—C1—C2—C3	−176.38 (17)	S2—C16—C17—C18	178.99 (15)
C6—C1—C2—C14	−178.5 (2)	C21—C16—C17—C29	179.6 (2)
S1—C1—C2—C14	3.4 (3)	S2—C16—C17—C29	−1.2 (3)
C1—C2—C3—C4	−0.5 (4)	C16—C17—C18—C19	−0.5 (3)
C14—C2—C3—C4	179.7 (3)	C29—C17—C18—C19	179.7 (2)
C2—C3—C4—C5	−0.5 (4)	C17—C18—C19—C20	0.1 (4)
C3—C4—C5—C6	0.2 (4)	C18—C19—C20—C21	0.9 (4)
C4—C5—C6—C1	1.1 (4)	C19—C20—C21—C16	−1.5 (3)
C2—C1—C6—C5	−2.1 (3)	C17—C16—C21—C20	1.2 (3)
S1—C1—C6—C5	176.18 (18)	S2—C16—C21—C20	−178.02 (16)
S1—N1—C7—O3	−1.4 (3)	S2—N2—C22—O6	−5.3 (3)
S1—N1—C7—C8	175.96 (13)	S2—N2—C22—C23	176.83 (13)
O3—C7—C8—C13	167.5 (2)	O6—C22—C23—C24	−15.9 (3)
N1—C7—C8—C13	−9.7 (3)	N2—C22—C23—C24	161.89 (18)
O3—C7—C8—C9	−10.1 (3)	O6—C22—C23—C28	163.0 (2)
N1—C7—C8—C9	172.63 (17)	N2—C22—C23—C28	−19.2 (3)
C13—C8—C9—C10	0.0 (3)	C28—C23—C24—C25	1.6 (3)
C7—C8—C9—C10	177.76 (17)	C22—C23—C24—C25	−179.42 (19)
C8—C9—C10—C11	−1.5 (3)	C23—C24—C25—C26	−1.1 (4)
C9—C10—C11—C12	1.2 (3)	C24—C25—C26—C27	−0.6 (4)
C9—C10—C11—C15	−178.4 (2)	C24—C25—C26—C30	−179.1 (2)
C10—C11—C12—C13	0.6 (4)	C25—C26—C27—C28	1.8 (4)
C15—C11—C12—C13	−179.9 (2)	C30—C26—C27—C28	−179.7 (2)
C11—C12—C13—C8	−2.0 (3)	C26—C27—C28—C23	−1.2 (4)
C9—C8—C13—C12	1.7 (3)	C24—C23—C28—C27	−0.5 (3)
C7—C8—C13—C12	−175.89 (19)	C22—C23—C28—C27	−179.4 (2)

### Hydrogen-bond geometry (Å, °)

**Table:**

*D*—H···*A*	*D*—H	H···*A*	*D*···*A*	*D*—H···*A*
N1—H1N···O4^i^	0.82 (2)	2.17 (2)	2.978 (2)	165 (2)
N2—H2N···O2^i^	0.83 (2)	2.20 (2)	3.022 (2)	171 (2)

Symmetry codes: (i) −*x*+1, −*y*, −*z*.

## References

[bb1] Gowda, B. T., Foro, S., Suchetan, P. A. & Fuess, H. (2009). *Acta Cryst.* E**65**, o2516. 10.1107/S1600536809037222 PMC2970249 21577963

[bb2] Gowda, B. T., Foro, S., Suchetan, P. A. & Fuess, H. (2010). *Acta Cryst.* E**66**, o433. 10.1107/S1600536810002254 PMC2979884 21579848

[bb3] Oxford Diffraction (2009). *CrysAlis CCD* and *CrysAlis RED* Oxford Diffraction Ltd, Yarnton, England.

[bb4] Sheldrick, G. M. (2008). *Acta Cryst.* A**64**, 112–122. 10.1107/S0108767307043930 18156677

[bb5] Spek, A. L. (2009). *Acta Cryst.* D**65**, 148–155. 10.1107/S090744490804362X PMC2631630 19171970

[bb6] Suchetan, P. A., Gowda, B. T., Foro, S. & Fuess, H. (2010). *Acta Cryst.* E**66**, o327. 10.1107/S1600536809055585 PMC2979700 21579757

